# Associations of lamplight exposure during sleep and sleep duration with attention-deficit/hyperactivity disorder among preschool children in China

**DOI:** 10.3389/fpsyt.2024.1489229

**Published:** 2025-01-08

**Authors:** Hui Jiang, Bin Yu, Yuanyang Liu, Hui Gao, Ruijuan Song, Siyue Tan, Shufen Han, Hui Zuo

**Affiliations:** ^1^ School of Public Health, Suzhou Medical College of Soochow University, Suzhou, China; ^2^ Medical Research Center, Sichuan Bingzhe Technology Co., Ltd, Chengdu, China; ^3^ School of Public Health, Hangzhou Normal University, Hangzhou, Zhejiang, China; ^4^ Jiangsu Key Laboratory of Preventive and Translational Medicine for Major Chronic Non-communicable Diseases, Suzhou Medical College of Soochow University, Suzhou, China; ^5^ Ministry of Education (MOE) Key Laboratory of Geriatric Diseases and Immunology, Suzhou Medical College of Soochow University, Suzhou, China

**Keywords:** lamplight exposure during sleep, sleep duration, ADHD, preschool children, China

## Abstract

**Introduction:**

This study aimed to examine the associations of exposure to light while sleeping at night and different sleep durations with attention-deficit/hyperactivity disorder (ADHD) among preschool children in China.

**Methods:**

A cross-sectional study including 4197 preschool children (2190 boys and 2007 girls) was conducted in 2021. Lamplight exposure during sleep and sleep duration were collected via a validated questionnaire. ADHD was assessed using the Conners Parental Symptom Questionnaire (PSQ). Associations of exposure to light while sleeping and sleep duration with ADHD were examined by multivariable logistic regression models.

**Results:**

The overall prevalence of ADHD was 3.7%. Compared to the children who slept with the lamplight off at night, those who slept with the lamplight on ≥3 nights/week were more likely to have ADHD after multivariable adjustment (OR=3.37; 95% CI: 1.95, 5.82; *P*-trend <0.001). The risk associations of lamplight exposure during sleep with ADHD were similar in subgroups stratified by sex, picky eating, gestational hyperglycemia, and gestational anemia. Moreover, compared to the children with sleep duration of 10-12 hours/day, adjusted OR for ADHD was 1.64 (95% CI: 1.14, 2.35) for those with sleep duration <10 hours/day and 2.69 (95% CI: 1.12, 6.44) for those who slept>12 hours/day.

**Discussion:**

Lamplight exposure during sleep at night was positively associated with ADHD among preschool children. Also, both sleep duration of <10 hours/day and >12 hours/day increased the likelihood of ADHD. Our novel findings suggest the importance of sleeping habit on the prevention of ADHD. Prospective and interventional studies are warranted to elucidate the observed associations.

## Introduction

Attention-deficit/hyperactivity disorder (ADHD), as a neurodevelopmental disorders, is a group of syndromes characterized by obvious inattention, hyperactivity and impulsivity (1). The prevalence of ADHD was about 7% and more than 1 million children were affected worldwide (2). ADHD is often accompanied by cognitive impairment and learning difficulties, including executive dysfunction, bad school performance, poor family and peer relationships, and aggressive behavior. It was reported that the incidence of ADHD co-morbid sleep disorders was 25% to 55% in youth (3). The main manifestations are bedtime resistance, sleep difficulty, night awakenings, sleep apnea, and daytime sleepiness (4). Although ADHD symptoms usually appear in childhood, it may continue to adulthood (5, 6), which can seriously affect their quality of life, bringing a huge burden to patients, families and society.

ADHD has been attributed to genetic factors (7) and family factors (8). Notably, with the development of industrial lighting, some people are accustomed to sleeping with lamplight, whereas visible light could have adverse effects on sleep, retina function and psychiatric disorders (9, 10).

No published studies, however, have explored the association between lamplight exposure during sleep at night and ADHD. In addition, previous studies showed that short sleep duration increased the risk of ADHD (11, 12), but the association of long sleep duration with ADHD remains unclear (13–15). Thus, the aim of the present study was to examine the associations of lamplight exposure during sleep and different sleep durations with ADHD among preschool children in China.

## Materials and methods

### Study participants

From October through December 2021, a cross-sectional survey was conducted in Chengdu, a provincial capital in Southwest China. It was mainly designed to assess the prevalence of and risk factors for psychobehavioral problems among preschool children. A total of 4360 preschool children from 30 kindergartens, were selected by cluster random sampling method. The study protocol was approved by the Ethics Committee of Soochow University (Approval NO. SUDA20210820H01). Written informed consent was obtained from each of the participants (guardians).

In the present study, we excluded 129 children aged less than 3 years and 34 children with missing information. Therefore, the final study population was 4197 children (2190 boys and 2007 girls, mean age 4.28 ± 0.93 years).

### Assessment of lamplight exposure during sleep and sleep duration

Data were collected by a validated questionnaire administered by guardians via WJX (an online survey platform, www.wjx.cn). Information on two independent variables was obtained by the following questions: “How often did your child sleep with the lamplight on at night? (a) 0 night/week, (b) 1-2 nights/week, (c) ≥3 nights/week”, and “For how long did the child sleep daily including siesta? (a) <10 hours/day, (b) 10-12 hours/day, (c) >12 hours/day”. According to the National Sleep Foundation’s sleep duration recommendation (16), total sleep duration with <10 hours/day for preschool children was defined as insufficient sleep in our study.

### Definition of ADHD

ADHD was screened using the Conners Parental Symptom Questionnaire (PSQ), which was compiled originally by the American scholar Conners and revised by Conners, Goyette, and Ulrich in 1978 (17). The PSQ has been widely applied to evaluate psychobehavioral problems of children and adolescents aged 3-17 years. It has been introduced or amended by many countries and shown high reliability and validity (18–20). Cronbach’s α value was 0.933 in our survey.

The 1978 version of PSQ was used in the present study, which included a total of 48 items and 6 measurement dimensions (i.e., conduct problems, learning problems, psychosomatic disorders, hyperactivity-impulsivity, anxiety, hyperactivity index). Hyperactivity index including 10 items was a special screening tool for ADHD. Participants were asked to rate their child’s behavior performance in the past six months. Each item was scored from 0 (not at all) to 3 (very often). ADHD was defined as having an average score of hyperactivity index of ≥1.5.

### Covariates

The children’s demographic characteristics including age (continuous), sex (male or female), lifestyle and family factors including snacks intake (0 days/week, 1-2 days/week, 3-4 days/week or ≥5 days/week), picky eating (yes or no), time of watching TV (0 hours/day, <1 hours/day, 1-2 hours/day or >2 hours/day), home lighting (dark, ordinary, or bright) were collected via the questionnaire. In addition, perinatal factors of the child’s mother including smoking or passive smoking during pregnancy (0 days/week, 1-2 days/week or ≥3 days/week), exercise during pregnancy (0 minutes/day, <20 minutes/day, 20-40 minutes/day, or >40 minutes/day), gestational hyperglycemia (yes or no), gestational hypertension (yes or no), gestational anemia (yes or no), and anxiety or depression during pregnancy (yes or no) were also collected.

### Statistical analyses

The basic characteristics of the participants by ADHD status, lamplight exposure during sleep at night and different sleep durations are presented as numbers (percentages), and were compared using Chi-square test or Fisher’s exact test.

We used logistic regression to evaluate the associations of lamplight exposure during sleep at night and sleep duration with ADHD. Three multivariable models were constructed: model 1 was the basic model adjusted for age and sex; model 2 was adjusted as model 1 plus the child’s lifestyle and family factors; model 3 was adjusted as model 2 plus the perinatal factors of mothers. *P*-trend was calculated by modeling the independent variables as continuous ones in the regression models.

Effect modification of the association between lamplight exposure during sleep and ADHD by sex, picky eating, gestational hyperglycemia and gestational anemia was estimated in stratified analyses, and significance of interactions was evaluated on first-degree multiplicative models for each stratification variable separately.

The statistical analyses were performed using SPSS 26.0 statistical software (IBM Corp., USA). All tests were two-tailed, and a *P* value <0.05 was considered statistically significant.

## Results

Of the 4197 preschool children, 154 (3.7%) had ADHD. The percentage of the participants who slept with lamplight 0, 1-2, and ≥3 nights/week was 91.5%, 4.7%, and 3.8%, respectively. The proportion of the participants whose sleep duration of <10, 10-12, and >12 hours/day was 24.6%, 72.7%, and 2.7%, respectively.

**Table 1** shows the characteristics of the participated children and their mothers by ADHD status. The boys were more likely to have ADHD than the girls. Those children dwelling in an environment without bright light, having frequent snacks intake, picky eating, and watching TV for a long time were more likely to have ADHD (all *P*< 0.001). Moreover, the prevalence of ADHD was higher in the children whose mother with smoking or passive smoking during pregnancy, less exercise during pregnancy, and gestational anemia (all *P*< 0.001).

**Table 1:** Characteristics of the participants according to ADHD status.

Characteristics	Overall	Non-ADHD	ADHD	*P Value*
Participants, n (%)	4197 (100)	4043 (96.3)	154 (3.7)	
Age (y)				
3	998 (23.8)	960 (23.7)	38 (24.7)	0.207
4	1405 (33.5)	1360 (33.6)	45 (29.2)	
5	1420 (33.8)	1358 (33.6)	62 (40.3)	
6	374 (8.9)	365 (9.1)	9 (5.8)	
Sex (%)				
Male	2190 (52.2)	2084 (51.5)	106 (68.8)	<0.001
Female	2007 (47.8)	1959 (48.5)	48 (31.2)	
Home lighting (%)				
Dark	142 (3.4)	126 (3.1)	16 (10.4)	<0.001
Ordinary	1989 (47.4)	1906 (47.1)	83 (53.9)	
Bright	2066 (49.2)	2011 (49.8)	55 (35.7)	
Snacks intake (days/week)				
0	506 (12.1)	499 (12.4)	7 (4.5)	<0.001
1-2	2287 (54.5)	2221 (54.9)	66 (42.9)	
3-4	876 (20.8)	830 (20.5)	46 (29.9)	
≥5	528 (12.6)	493 (12.2)	35 (22.7)	
Picky eating (%)				
Yes	2045 (48.7)	1936 (47.9)	109 (70.8)	<0.001
No	2152 (51.3)	2107 (52.1)	45 (29.2)	
Time of watching TV (hours/day)				
0	1056 (25.2)	1025 (25.4)	31 (20.1)	<0.001
<1	1937 (46.2)	1877 (46.4)	60 (39.0)	
1-2	1012 (24.1)	972 (24.0)	40 (26.0)	
>2	192 (4.5)	169 (4.2)	23 (14.9)	
Smoking or passive smoking during pregnancy (days/week)				
0	3918 (93.4)	3789 (93.7)	129 (83.8)	<0.001
1-2	179 (4.3)	165 (4.1)	14 (9.1)	
≥3	100 (2.3)	89 (2.2)	11 (7.1)	
Exercise during pregnancy (minutes/day)				
0	284 (6.8)	262 (6.5)	22 (14.3)	<0.001
<20	644 (15.3)	608 (15.0)	36 (23.4)	
20-40	1771 (42.2)	1721 (42.6)	50 (32.5)	
>40	1498 (35.7)	1452 (35.9)	46 (29.8)	
Gestational hyperglycemia (%)				
Yes	570 (13.6)	548 (13.6)	22 (14.3)	0.795
No	3627 (86.4)	3495 (86.4)	132 (85.7)	
Gestational hypertension (%)				
Yes	151 (3.6)	145 (3.6)	6 (3.9)	0.840
No	4046 (96.4)	3898 (96.4)	148 (96.1)	
Gestational anemia (%)				
Yes	1012 (24.1)	956 (23.6)	56 (36.4)	<0.001
No	3185 (75.9)	3087 (76.4)	98 (63.6)	
Anxiety or depression during pregnancy (%)				
Yes	110 (2.6)	103 (2.5)	7 (4.5)	0.205
No	4087 (97.4)	3940 (97.5)	147 (95.5)	
Lamplight exposure during sleep (nights/week)				
0	3839 (91.5)	3716 (91.9)	123 (79.9)	<0.001
1-2	197 (4.7)	184 (4.6)	13 (8.4)	
≥3	161 (3.8)	143 (3.5)	18 (11.7)	
Sleep duration including siesta (hours/day)				
<10	1033 (24.6)	977 (24.2)	56 (36.4)	0.001
10-12	3049 (72.6)	2957 (73.1)	92 (59.7)	
>12	115 (2.8)	109 (2.7)	6 (3.9)	

Data are presented as numbers (percentages). *P* values were determined by Chi-square test or Fisher's exact test.

The characteristics of the participants in term of lamplight exposure during sleep at night are presented in **Table 2**. Percentages of picky eating, time of watching TV and proportion of gestational anemia were different between the groups (*P*< 0.05). Moreover, age, home lighting, picky eating, time of watching TV, exercise during pregnancy, and anxiety or depression during pregnancy were also different with different sleep durations (*P*< 0.05) (**Supplementary Table S1**).

**Table 2:** Characteristics of the participants according to lamplight exposure during sleep.

Characteristics	Lamplight exposure during sleep (nights/week)			*P Value*
	0	1-2	≥3	
Participants, n (%)	3839 (91.5)	197 (4.7)	161 (3.8)	
Age (y)				
3	902 (23.5)	44 (22.3)	52 (32.3)	0.182
4	1292 (33.7)	66 (33.5)	47 (29.2)	
5	1308 (34.0)	64 (32.5)	48 (29.8)	
6	337 (8.8)	23 (11.7)	14 (8.7)	
Sex (%)				
Male	1999 (52.1)	104 (52.8)	87 (54.0)	0.874
Female	1840 (47.9)	93 (47.2)	74 (46.0)	
Home lighting (%)				
Dark	127 (3.3)	11 (5.6)	4 (2.5)	0.121
Ordinary	1805 (47.0)	104 (52.8)	80 (49.7)	
Bright	1907 (49.7)	82 (41.6)	77 (47.8)	
Snacks intake (days/week)				
0	475 (12.4)	16 (8.1)	15 (9.3)	0.071
1-2	2102 (54.8)	109 (55.3)	76 (47.2)	
3-4	786 (20.4)	47 (23.9)	43 (26.7)	
≥5	476 (12.4)	25 (12.7)	27 (16.8)	
Picky eating (%)				
Yes	1847 (48.1)	107 (54.3)	91 (56.5)	0.031
No	1992 (51.9)	90 (45.7)	70 (43.5)	
Time of watching TV (hours/day)				
0	979 (25.5)	39 (19.8)	38 (23.6)	0.015
<1	1773 (46.2)	100 (50.8)	64 (39.8)	
1-2	923 (24.0)	43 (21.8)	46 (28.6)	
>2	164 (4.3)	15 (7.6)	13 (8.0)	
Smoking or passive smoking during pregnancy (days/week)				
0	3590 (93.5)	181 (91.9)	147 (91.3)	0.422
1-2	157 (4.1)	12 (6.1)	10 (6.2)	
≥3	92 (2.4)	4 (2.0)	4 (2.5)	
Exercise during pregnancy (minutes/day)				
0	256 (6.7)	14 (7.1)	14 (8.7)	0.218
<20	577 (15.0)	34 (17.3)	33 (20.5)	
20-40	1622 (42.3)	90 (45.7)	59 (36.6)	
>40	1384 (36.0)	59 (29.9)	55 (34.2)	
Gestational hyperglycemia (%)				
Yes	511 (13.3)	31 (15.7)	28 (17.4)	0.222
No	3328 (86.7)	166 (84.3)	133 (82.6)	
Gestational hypertension (%)				
Yes	137 (3.6)	9 (4.6)	5 (3.1)	
No	3702 (96.4)	188 (95.4)	156 (96.9)	0.720
Gestational anemia (%)				
Yes	903 (23.5)	66 (33.5)	43 (26.7)	0.004
No	2936 (76.5)	131 (66.5)	118 (73.3)	
Anxiety or depression during pregnancy (%)				
Yes	100 (2.6)	4 (2.0)	6 (3.7)	0.539
No	3739 (97.4)	193 (98.0)	155 (96.3)	

Data are presented as numbers (percentages). *P* values were determined by Chi-square test or Fisher's exact test.

As shown in **Table 3**, compared with the children who slept with the lamplight off, the multivariable adjusted OR was 1.71 (95% CI: 0.92, 3.21) and 3.37 (95% CI: 1.95, 5.82) for those who slept with the lamplight on 1-2 nights/week, and those who slept with the lamplight on ≥3 nights/week, respectively (*P*-trend <0.001).

**Table 3:** ORs (95% CI) for lamplight exposure during sleep and sleep duration in association with ADHD among the preschool children.

	Cases/n	Model 1	Model 2	Model 3
Lamplight exposure during sleep (nights/week)				
0	123/3839	1.00 (Reference)	1.00 (Reference)	1.00 (Reference)
1-2	13/197	2.13 (1.18, 3.86)	1.75 (0.95, 3.24)	1.71 (0.92, 3.21)
≥3	18/161	3.79 (2.24, 6.41)	3.39 (1.98, 5.83)	3.37 (1.95, 5.82)
*P*-trend		<0.001	<0.001	<0.001
Sleep duration (hours/day)				
<10	56/1033	1.89 (1.34, 2.67)	1.74 (1.22, 2.50)	1.64 (1.14, 2.35)
10-12	92/3049	1.00 (Reference)	1.00 (Reference)	1.00 (Reference)
>12	6/115	1.90 (0.81, 4.45)	2.57 (1.08, 6.11)	2.69 (1.12, 6.44)

Model 1 was adjusted for age and sex.

Model 2 was adjusted for model 1 plus home lighting, snacks intake, picky eating, and time of watching TV.

Model 3 was adjusted for model 2 plus smoking or passive smoking during pregnancy, exercise during pregnancy,gestational hyperglycemia, gestational hypertension, gestational anemia, and anxiety or depression during pregnancy.

Compared with those who slept for 10-12 hours/day, the children who slept less than 10 hours/day had higher odds of ADHD (multivariable-adjusted OR=1.64; 95% CI: 1.14, 2.35), similar with those with sleep duration >12 hours/day (multivariable-adjusted OR=2.69; 95% CI: 1.12, 6.44) (**Table 3**).

**Figure 1:**
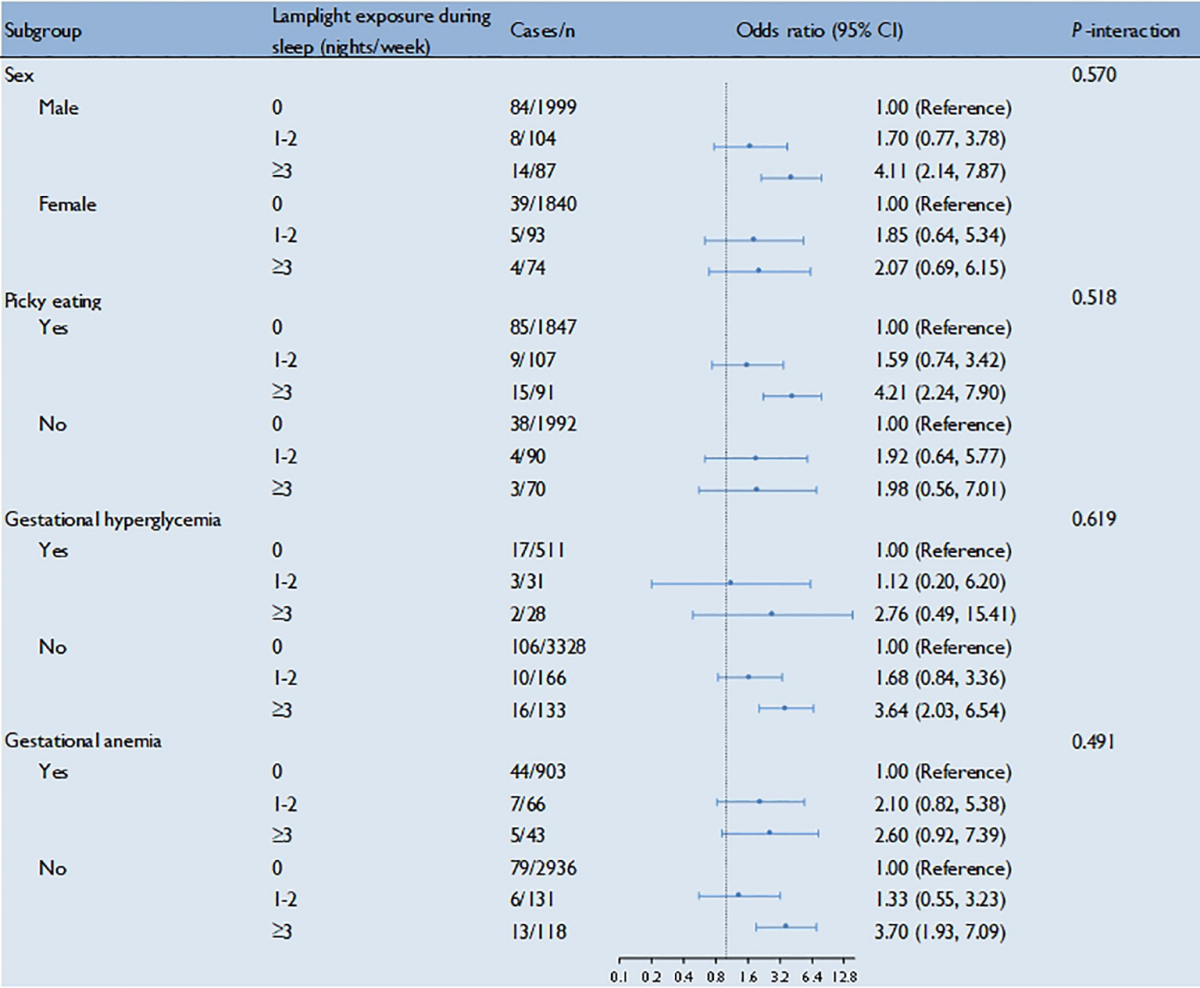
Lamplight exposure during sleep in association with ADHD among the preschool children by strata. Multivariable adjusted model: adjusted for age, sex, home lighting, snacks intake, picky eating, time of watching TV, smoking or passive smoking during pregnancy, exercise during pregnancy, gestational hyperglycemia, gestational hypertension, gestational anemia, and anxiety or depression during pregnancy.

The associations between lamplight exposure during sleep and ADHD were similar in subgroups stratified by sex, picky eating, gestational hyperglycemia, and gestational anemia at baseline (all *P*-interaction > 0.05) (**Figure 1**).

## Discussion

### Principal findings

In this cross-sectional study, we observed that lamplight exposure during sleep at night was associated with increased odds of ADHD among the preschool children. Moreover, both sleep duration of <10 hours/day and >12 hours/day increased the likelihood of ADHD as well.

### Lamplight exposure during sleep at night, sleep duration and ADHD

Accumulating evidence supports that exposure to lamplight (indoor and outdoor light) in the evening is related to various adverse health problems including psychobehavioral disorders (21–24). Outdoor light exposure at night was significantly associated with a higher risk of autism spectrum disorder (ASD, a neurodevelopmental disorder) in children (24); nighttime exposure to light could be able to induce neurological changes, mood disorders and depressive-like behaviors (21–24).

In our study, insufficient sleep duration increased odds of ADHD, which was consistent with previous studies (11, 12). Long sleep duration also increased the likelihood of ADHD, which was supported by Bogdan’s findings (13), but inconsistent with Lee’s (17). The inconsistency may come from different study design, screening tools for ADHD, classification of sleep duration, sample size and characteristics of the study populations.

### Possible mechanisms

The underlying mechanisms for the association of lamplight exposure during sleep with ADHD remain unclear. However, we proposed several potential explanations.

First, nighttime light can directly affect cognition and mood through intrinsically photosensitive retinal ganglion cells (ipRGC) projections to brain regions involved in emotional regulation (25). Second, nighttime light can indirectly affect mood and behaviors by altering clock and inflammation genes expression, hormone secretion, neuroplasticity and neurotransmission (22). Third, nighttime lamplight is able to disturb the circadian rhythm, inhibit the secretion of melatonin, directly stimulate brain activity (26, 27), and consequently trigger sleep disorders, which have been associated with the risk of ADHD (28, 29). Additionally, studies have demonstrated that light exposure can alter gene expression linked to (neuro)development, including crf, crfbp, mr, and gr-alpha (30). Crf antagonists improved the ability to work memory, the same as all approved ADHD treatments (31). This suggests that nighttime lamplight exposure in preschool children might raise ADHD risk potentially via crf gene regulation.

In this study, we also observed positive associations of both short and long sleep duration with ADHD among the preschool children. Insufficient sleep could damage the prefrontal cortex of the brain and limit the link between the prefrontal cortex and the amygdala (32), thereby affecting cognition, emotion control and executive function (33), which may explain the neurobehavioral, neurocognitive, and functional manifestations of ADHD symptomatology (28). The underlying mechanisms for the association of excessive sleep with ADHD remain unclear, the following mechanism may be involved: excessive sleep may increase arterial stiffness (34), reduce blood flow, and thus adversely affect the nervous system of the children.

### Strengths and limitations

To the best of our knowledge, this is the first study to examine the association between lamplight exposure during sleep and ADHD. Other main strengths include a relatively large sample size and adjustment for a number of confounders in the regression models.

Nevertheless, the study has also several limitations. First, this study was based on a cross-sectional design, therefore causal and temporal relationships could not be inferred. Reverse causality (i.e. ADHD causes sleeping with the light on at night and changed sleeping duration) can not be precluded. Researchers should confirm causality through longitudinal study and experimental study in the future. Second, based on the PSQ scale, preschool children with a hyperactivity index greater than 1.5 were classified as ADHD in this study. This may have resulted in misclassification. To address this limitation, future studies should consider using clinically diagnosed ADHD cases to enhance accuracy. Third, reporting bias and recall bias might have occurred due to self-report by guardians of the children. Future studies should prefer using objective measures (e.g., actigraphy for sleep). Fourth, although we have adjusted for a number of confounders, residual confounding by unknown or unmeasured factors including other sleep disorders cannot be ruled out. For example, children with low socioeconomic status may have a greater likelihood of ADHD (35). A central disorder of hypersomnolence may cause prolonged nighttime sleepiness, and studies have shown that ADHD occurs with a high frequency in adults with this condition (36). Fifth, our study focused on preschool children in Chengdu city, which may have restricted generalizability of the results. Therefore, future studies from different geographic areas or age groups are warranted.

## Conclusions

In the present study, nighttime lamplight exposure was positively associated with ADHD among the preschool children in China. And also, both insufficient and long sleep duration were associated with increased odds of ADHD. Although longitudinal or intervention-based studies are needed, our findings underline the importance of keeping healthy sleep habits for ADHD prevention in children. Specifically, we proposes two recommendations to reduce the risk of ADHD in children. First, the sleep duration of preschool children should be within an appropriate range. Second, it is necessary to provide children a light-free sleep environment by turning off the light, drawing curtains, avoiding electronic screen before bed, and staying away from electronic products.

## Data Availability

The raw data supporting the conclusions of this article will be made available by the authors, without undue reservation.

## Supplementary material

Supplementary Table 1 Characteristics of the participants according to different sleep durations.
