# Supplementary material for: Associations of lamplight exposure during sleep and sleep duration with attention-deficit/hyperactivity disorder among preschool children in China
Source: Front Psychiatry. 2025 Jan 8;15:1489229. doi: 10.3389/fpsyt.2024.1489229 (PMC11751368; doi:10.3389/fpsyt.2024.1489229)
Supplement: Supplementary Table 1 — Characteristics of the participants according to different sleep durations. [file Table1.docx]

| **Supplemental Table 1.** Characteristics of the participants according to different sleep durations | | | | | |
| --- | --- | --- | --- | --- | --- |
| Characteristics | | Sleep duration (hours/day) | | | *P Value* |
|  |  | <10 | 10-12 | >12 |  |
| Participants, n (%) | | 1033 (24.6) | 3049 (72.6) | 115 (2.8) |  |
| Age (y) | |  |  |  |  |
|  | 3 | 179 (17.4) | 775 (25.4) | 44 (38.3) | <0.001 |
|  | 4 | 312 (30.2) | 1058 (34.7) | 35 (30.4) |  |
|  | 5 | 430 (41.6) | 967 (31.7) | 23 (20.0) |  |
|  | 6 | 112 (10.8) | 249 (8.2) | 13 (11.3) |  |
| Sex (%) | |  |  |  |  |
|  | Male | 530 (51.3) | 1611 (52.8) | 49 (42.6) | 0.079 |
|  | Female | 503 (48.7) | 1438 (47.2) | 66 (57.4) |  |
| Home lighting (%) | |  |  |  |  |
|  | Dark | 50 (4.8) | 91 (3.0) | 1 (0.9) | 0.001 |
|  | Ordinary | 503 (48.7) | 1444 (47.3) | 42 (36.5) |  |
|  | Bright | 480 (46.5) | 1514 (49.7) | 72 (62.6) |  |
| Snacks intake (days/week) | |  |  |  |  |
|  | 0 | 132 (12.8) | 354 (11.6) | 20 (17.4) | 0.281 |
|  | 1-2 | 563 (54.5) | 1660 (54.4) | 64 (55.7) |  |
|  | 3-4 | 218 (21.1) | 642 (21.1) | 16 (13.9) |  |
|  | ≥5 | 120 (11.6) | 393 (12.9) | 15 (13.0) |  |
| Picky eating (%) | |  |  |  |  |
|  | Yes | 558 (54.0) | 1430 (46.9) | 57 (49.6) | <0.001 |
|  | No | 475 (46.0) | 1619 (53.1) | 58 (50.4) |  |
| Time of watching TV (hours/day) | |  |  |  |  |
|  | 0 | 241 (23.3) | 781 (25.6) | 34 (29.6) | 0.042 |
|  | <1 | 465 (45.0) | 1419 (46.5) | 53 (46.1) |  |
|  | 1-2 | 265 (25.7) | 720 (23.6) | 27 (23.4) |  |
|  | >2 | 62 (6.0) | 129 (4.3) | 1 (0.9) |  |
| Smoking or passive smoking during pregnancy (days/week) | |  |  |  |  |
|  | 0 | 957 (92.6) | 2854 (93.6) | 107 (93.0) | 0.321 |
|  | 1-2 | 44 (4.3) | 128 (4.2) | 7 (6.1) |  |
|  | ≥3 | 32 (3.1) | 67 (2.2) | 1 (0.9) |  |
| Exercise during pregnancy (minutes/day) | |  |  |  |  |
|  | 0 | 87 (8.4) | 193 (6.3) | 4 (3.5) | <0.001 |
|  | <20 | 219 (21.2) | 413 (13.5) | 12 (10.4) |  |
|  | 20-40 | 413 (40.0) | 1314 (43.2) | 44 (38.3) |  |
|  | >40 | 314 (30.4) | 1129 (37.0) | 55 (47.8) |  |
| Gestational hyperglycemia (%) | |  |  |  |  |
|  | Yes | 135 (13.1) | 421 (13.8) | 14 (12.2） | 0.756 |
|  | No | 898 (86.9) | 2628 (86.2) | 101 (87.8) |  |
| Gestational hypertension (%) | |  |  |  |  |
|  | Yes | 42 (4.1) | 101 (3.3) | 8 (7.0) | 0.080 |
|  | No | 991 (95.9) | 2948 (96.7) | 107 (93.0) |  |
| Gestational anemia (%) | |  |  |  |  |
|  | Yes | 252 (24.4) | 724 (23.7) | 36 (31.3) | 0.172 |
|  | No | 781 (75.6) | 2325 (76.3) | 79 (68.7) |  |
| Anxiety or depression during pregnancy (%) | |  |  |  |  |
|  | Yes | 40 (3.9) | 65 (2.1) | 5 (4.3) | 0.004 |
|  | No | 993 (96.1) | 2984 (97.9) | 110 (95.7) |  |
| Data are presented as numbers (percentages). *P* values were determined by Chi-square test or Fisher's exact test. | | | | | |
